# Access to mifepristone, misoprostol, and contraceptive medicines in eight countries in the Eastern Mediterranean Region: descriptive analyses of country-level assessments

**DOI:** 10.1186/s12978-024-01805-1

**Published:** 2024-06-04

**Authors:** Mohamed Afifi, Nilmini Hemachandra, Qais Sikandar, Rana Hajjeh, Ulrika Rehnström Loi, Laurence Läser, Dima Qato, Zahir Sidiqui Abdul, Paata Chikvaidze, Raghad Abdul Redha Abbas, Khalid Al-Kinani, Hanan Hasan, Faysal El-Kak, Alissar Rady, Omelkheir Brngali, Mohamed Hashem, Rachid Bezad, Cheikh Amine, Hachri Hafid, Sabeen Afzal, Raza Zaidi, Ellen Thom, Qudsia Uzma, Hadeel Al-Masri, Zakri Abu Qamar, Buthaina Ghanem, Itimad Abu Ward, Ali Nashat Shaar, Ubah Farah, Yusuf Omar Mohamed, Al-Umra Umar, Maha Eladawy

**Affiliations:** 1 World Health Organization, Regional Office for the Eastern Mediterranean, Cairo, Egypt; 2 Development and Research Training in Human Reproduction (HRP), Department of Sexual and Reproductive Health and Research, World Health Organization, UNDP-UNFPA-UNICEF-WHO-World Bank Special Programme of Research, Geneva, Switzerland; 3 Ministry of Public Health, Kabul, Afghanistan; 4 World Health Organization, Afghanistan Country Office, Kabul, Afghanistan; 5 Ministry of Health, Baghdad, Iraq; 6 University of Baghdad, Baghdad, Iraq; 7 World Health Organization, Iraq Country Office, Baghdad, Iraq; 8 American University in Beirut, Beirut, Lebanon; 9 Lebanon Country Office, World Health Organization, Beirut, Lebanon; 10 Ministry of Health, Tripoli, Libya; 11 World Health Organization, Libya Country Office, Tripoli, Libya; 12 Mohammed V University of Rabat, Rabat, Morocco; 13 Cheikh Zaid Hospital, Rabat, Morocco; 14 World Health Organization, Morocco Country Office, Rabat, Morocco; 15 Ministry of National Health Services Regulations and Coordination, Islamabad, Pakistan; 16 Pakistan Country Office, World Health Organization, Islamabad, Pakistan; 17 Palestinian Ministry of Health, Ramallah, Palestine; 18 World Health Organization Office for West Bank and Gaza, Jerusalem, Palestine; 19 An-Najah National University, West Bank, Palestine; 20 Ministry of Health and Human Services, Mogadishu, Somalia; 21 Somalia Country Office, World Health Organization, Mogadishu, Somalia

**Keywords:** Contraceptives, Mifepristone, Misoprostol, Essential medicines list, Sexual and reproductive health

## Abstract

**Background:**

Despite their importance in reducing maternal mortality, information on access to Mifepristone, Misoprostol, and contraceptive medicines in the Eastern Mediterranean Region is limited.

**Methods:**

A standardized assessment tool measuring access to Mifepristone, Misoprostol, and contraceptive medicines included in the WHO essential medicines list (EML) was implemented in eight countries in the Eastern Mediterranean Region (Afghanistan, Iraq, Lebanon, Libya, Morocco, Palestine, Pakistan, and Somalia) between 2020–2021. The assessment focused on five access measures: 1) the inclusion of medicines in national family planning guidelines; 2) inclusion of medicines in comprehensive abortion care guidelines; 3) inclusion of medicines on national essential medicines lists; 4) medicines registration; and 5) procurement and forecasting of Mifepristone, Misoprostol, and contraceptive medicines. A descriptive analysis of findings from these eight national assessments was conducted.

**Results:**

Only Lebanon and Pakistan included all 12 contraceptives that are enlisted in the WHO-EML within their national family planning guidelines. Only Afghanistan and Lebanon included mifepristone and mifepristone-misoprostol combination in post-abortion care guidelines, but these medicines were not included in their national EMLs. Libya and Somalia lacked a national regulatory authority for medicines registration. Most contraceptives included on the national EMLs for Lebanon, Morocco and Pakistan were registered. Misoprostol was included on the EMLs—and registered—in six countries (Afghanistan, Iraq, Lebanon, Morocco, Palestine, and Pakistan). However, only three countries procured misoprostol (Iraq, Morocco, and Somalia).

**Conclusion:**

These findings can guide efforts aimed at improving the availability of Mifepristone, Misoprostol, and contraceptive medicines in the Eastern Mediterranean Region. Opportunities include expanding national EMLs to include more options for Mifepristone, Misoprostol, and contraceptive medicines and strengthening the registration and procurement systems to ensure these medicines’ availability were permitted under national law and where culturally acceptable.

**Supplementary Information:**

The online version contains supplementary material available at 10.1186/s12978-024-01805-1.

## Abstract (plain language summary)

Ensuring access to Mifepristone, Misoprostol, and contraceptive medicines is critical to improving women’s health, and more specifically reducing maternal mortality and improving women’s sexual and reproductive health in the Eastern Mediterranean Region.

The aim of this study was to analyse findings from national assessments to capture information on the implementation of relevant policies and procedures. Those were the policies that ensure access to Mifepristone, Misoprostol, and contraceptive medicines in the public sector for the eight Eastern Mediterranean Region countries included in the study (Afghanistan, Iraq, Libya, Lebanon, Morocco, Palestine, Pakistan, and Somalia). The assessments were completed between 2020 and 2021.

We found that most countries did not include all twelve contraceptives enlisted in the WHO essential medicines list (EML) in their national family planning guidelines. No country had developed a national abortion care guidelines nor included mifepristone (alone or in combination with misoprostol) on national EML. Libya and Somalia lacked a national regulatory authority for medicines registration. Most contraceptives included on the national EMLs for Lebanon, Morocco and Pakistan were registered. Misoprostol was included on the EMLs—and registered—in six countries (Afghanistan, Iraq, Lebanon, Morocco, Palestine, and Pakistan) yet, only three countries procured misoprostol (Iraq, Morocco, and Somalia).

Our findings provide evidence on system-level barriers to availability of Mifepristone, Misoprostol, and contraceptive medicines (e.g., lack of guidelines or inclusion on EML, lack of registration and procurement) that can support policy and advocacy efforts to strengthen the pharmaceutical sector to better ensure availability of Mifepristone, Misoprostol, and contraceptive medicines to women in reproductive age at the country-level in accordance with the national law and prevailing culture.

**Supplementary Information:**

The online version contains supplementary material available at 10.1186/s12978-024-01805-1.

## Background

Ensuring access to Mifepristone, Misoprostol, and contraceptive medicines for women of reproductive age is critical to reducing maternal mortality and advancing sexual and reproductive health and reproductive rights (SRH&RR) [1, 2]. Access to contraception improves women’s health, prevents unintended pregnancies and decreases the occurrence of unsafe abortion. Abortion is safe when carried out using a method recommended by WHO, appropriate to the pregnancy duration and by someone with the necessary skills. However, when women with unwanted pregnancies face barriers to obtaining quality abortion, they often resort to unsafe abortion, which is a leading cause of preventable maternal deaths [3–5].

Countries in the Eastern Mediterranean Region (EMR) often rely on the World Health Organization (WHO) evidence-based guidelines and Model List of Essential Medicines when developing and updating their national guidelines and national essential medicines lists (EML). Despite the availability of WHO guidelines focusing on family planning, selected practice recommendations for contraceptive use [6] and comprehensive abortion care (CAC) [7], the extent to which EMR countries have developed similar guidelines is not documented. The inclusion of the twelve contraceptives and Mifepristone, Misoprostol, and Combipack on the WHO EML—which are necessary to implementing the WHO guidelines—on national EMLs in EMR countries, and information on the registration, procurement and forecasting of Mifepristone, Misoprostol, and contraceptives is not known.

This information is important for several reasons. First, it will provide an evidence-base by identifying system-level barriers and can support policy and advocacy efforts to strengthen the pharmaceutical sector, as well as better ensure availability of Mifepristone, Misoprostol, and contraceptives to women of reproductive age at the country-level in accordance with the national law and prevailing culture. Second, Mifepristone, Misoprostol, and contraceptives are critical in promoting SRH&RR and reducing maternal mortality [8–16], especially in settings that persistently have high maternal mortality rates [17]. Several EMR countries also are among the list with the lowest rates of contraceptive use and highest rates of unintended pregnancy [3, 8–10] Third, nearly all EMR countries have abortion care policies that permit it only to preserve a woman's life or health [4, 18]. Therefore, identifying barriers in availability and access to quality-assured Mifepristone, Misoprostol, and contraceptives is critical to addressing the burden of unintended pregnancy and unsafe abortions in these countries [19, 20].

To address this knowledge gap, the WHO Eastern Mediterranean Office (EMRO) developed a standardised tool for the assessment of the situation of essential medicines required to implement national SRH&RR guidelines (see Supplement 1). The goal of the assessment was to evaluate the implementation of policy and system level requirements necessary to ensure access to Mifepristone, Misoprostol, and contraceptives in these EMR countries. The assessments were completed between 2020 and 2021 in the following eight EMR countries: Afghanistan, Iraq, Libya, Lebanon, Morocco, Palestine, Pakistan, and Somalia. In this paper, we describe the findings from these country assessments and provide opportunities for improving access to Mifepristone, Misoprostol, and contraceptives at the regional and country-level.

## Methods

### Access measures

In 2020, the WHO EMRO developed a SRH essential medicines national assessment tool to measure access to essential medicines at the system level in the public sector encompassing five SRH areas (preconception care, maternal and newborn care, intrapartum and post-partum care, family planning/contraception, and safe abortion care) (see Additional File 1). The assessment tool captured information for the five access measures listed below. In this study, we focus on family planning/contraception and safe abortion care components of the assessment for each of the 15 essential medicines for contraception and safe abortion included on the WHO EML (see Table 2):

1. Guidelines: Inclusion of essential medicines in national guidelines or protocols for family planning, contraception and/or comprehensive abortion care.
2. EML: Inclusion of essential medicines on national EML
3. Registration: Essential medicines registered with National Regulatory Authority
4. Procurement: Inclusion of essential medicines in procurement list for public sector.
5. Forecasting: Inclusion of essential medicines in forecasting tools for medicines in the public sector

Countries from the EMR regions were invited to participate in this study and were selected based on a range of selection criteria including considering the issue under investigation a current priority, burden of disease, maternal and neonatal mortalities, willingness, and availability to participate within the planned timeframe. Ultimately, eight countries from the EMR (Afghanistan, Pakistan, Morocco, Iraq, Lebanon, Libya, Palestine, Somalia), agreed to participate in this study and were therefore included. The assessment tool was then shared with all eight countries and data collection was conducted by Ministry of Health staff and/or consultants in the country.

### Data collection

Data collection involved a mixed-methods approach to gather the necessary information to complete the assessment following guidance and procedures. This process included a desk review of available guidelines/protocols and policy documents, including national EMLs and procurement lists, and consultations with key policy and regulatory authorities, Ministry of Health departments, and program officials.

Data collected was then used to complete the assessment for the five access measures. For example, if national family planning guidelines were available, the medicines necessary to implement these guidelines were identified and then mapped to the assessment tool measure #1 Guidelines above. In addition, #2 national EMLs were reviewed, and specific contraceptives and Mifepristone and Misoprostol were identified. Further, whether contraceptives and Mifepristone and Misoprostol mentioned in guidelines or included on national EML were #3 registered for marketing authorization at a national regulatory authority (NRA), #4 included in procurement lists, and #5 forecasting tools, was also assessed.

### Analysis

Completed assessments were then reviewed and analyzed for each of the 15 essential medicines (of which 12 were contraceptives and three were medical abortion medicines) for all five access measures for each of the eight EMR countries. To interpret the findings from the assessment in the context of the country's situation, we also summarized information on the availability of national guidelines for family planning and/or comprehensive abortion care, including year of publication, year of current national EML used in the implementation of the assessment, and the legal landscape. We focused our analysis and reporting of results on the extent to which countries include these medicines in national guidelines and EMLs and whether they are registered and procured in the public sector for each country.

## Results

### Availability of national family planning guidelines, guidelines for comprehensive abortion care, essential medicines lists by country and legal landscape

As summarised in Table 1, five countries (Afghanistan, Iraq, Lebanon, Palestine, and Pakistan) had family planning guidelines or protocols available that were recently published between 2016–2018, whereas Morocco guidelines have not been updated since 2007. As of 2021, Post-abortion care (PAC) guidelines were only available in Afghanistan and Pakistan. Comprehensive abortion care (CAC) guidelines were not available in any country. Lebanon relies on Service Delivery Guidelines for Reproductive Health for both family planning and abortion care. National EML's were available for all eight countries with the most current being 2020–2021 (Iraq and Palestine). All other countries had national EMLs with the most recent updates around 2017. Morocco had the least restrictive abortion laws among these eight countries, where abortion was permitted to preserve health. All other countries only permit induced abortion to save a woman's life with exceptions for rape, incest and/or foetal abnormalities in some countries.

**Table 1:** Availability of Family Planning/Contraception and Abortion Care Guidelines, by Country

**COUNTRY**	**EML**	**FAMILY PLANNING GUIDELINES/PROTOCOL**	**ABORTION CARE GUIDELINES/PROTOCOL**	**ABORTION RESTRICTION**^a^
	**[YEAR]**	**[SOURCE, YEAR]**	**[SOURCE, YEAR]**	
**Afghanistan**	2017	National Family Planning Guidelines, 2017	Post Abortion Care Guidelines, 2017	To Save Woman's Life
**Iraq**	2020	National Family Planning Guidelines, 2018	Misoprostol Protocol, 2018	To Save Woman's Life
**Lebanon**	2018	Service Delivery Guidelines for Reproductive Health, 2016	Service Delivery Guidelines for Reproductive Health, 2016	To Save Woman's Life
**Libya**	-	SRHR Guidelines,	None	To Save Woman's Life
**Morocco**	2019	Standards for Family Planning Methods, 2007	-	To Preserve Health
**occupied Palestinian territory**	2021–2022	Family Planning Protocol, 2016–2017	None^b^	To Save Woman's Life^c^
**Pakistan**	2018	National Guidelines and Standards for Family Planning Services, 2017	National Service Delivery Standards and Guidelines for Post Abortion Care, 2018^d^	To Preserve Health
**Somalia**	2019	Somali Birth Spacing Guidelines 2008^e^	None	To Save Woman's Life

^a^https://abortion-policies.srhr.org/

^b^The occupied Palestinian territory does not have a national CAC or PAC guidelines, practitioners rely on international guidelines for PAC

^c^Exceptions in the case of rape, incest and severe fetal anomalies

^d^In Pakistan, the provinces have the autonomy to develop their own standards and guidelines on abortion and post-abortion care. So far, Punjab is the only province to have developed and adopted standards and guidelines on abortion and post-abortion care, in 2015: "Service Delivery Standards and Guidelines for High Quality Safe Uterine Evacuation and Postabortion Care" which (among other things) outline the role and level of providers authorized, trained and supported for the provision of safe uterine evacuation and postabortion care within Pakistan's legal framework. There are also national guidelines, such as the Pakistan Woman Centered Post Abortion Care Reference Manual, from 2015

^e^Somalia updated family planning guidelines and develop post-abortion care guidelines in December 2022

### Inclusion of WHO essential medicines for contraception and medical abortion in national guidelines or protocols

#### Contraceptives

Twelve essential medicines are listed on the current WHO EML that includes six categories/formulations of contraceptives (oral hormonal contraceptives, injectables, intrauterine devices (IUDs), implants, vaginal rings, and emergency contraceptives) (Table 2). All eight countries included ethinylestradiol-levonorgestrel, copper-containing IUDs, and levonorgestrel for emergency contraception in guidelines. All countries had at least one essential medicine from each contraceptive category mentioned in national guidelines. Lebanon and Pakistan included all 12 contraceptives in their guidelines. Importantly, all eight countries had the emergency contraceptive (levonorgestrel) mentioned in the guidelines.

**Table 2:** Inclusion of Essential Medicines in Family Planning and Abortion Guidelines, by Country

Essential Medicines for Contraception and Medical Abortion (MA) in National Family Planning and/or Abortion Care Guidelines or Protocols	AFGHANISTAN	IRAQ	LEBANON	LIBYA	MOROCCO	oPt	PAKISTAN	SOMALIA	Total No. of Countries
Oral Hormonal Contraceptives									
Ethinylestradiol (30ug) + levonorgestrel (150 ug)	✓	✓	✓	✓	✓	✓	✓	✓	**8**
Ethinylestradiol (35ug) + norethisterone (1 mg)	✓	✓	✓	✓	✗	✓	✓	✓	**7**
Injectables									
Medroxyprogesterone acetate IM/SC injection	✓	✓	✓	✓	✓	✓	✓	✓	**8**
Estradiol cypionate + medroxyprogesterone acetate (5-25 mg) inj	✓	✗	✓	✓	✗	✗	✓	✓	**5**
Norethisterone enantate: 200 mg/ mL in 1- mL ampoule	✓	✓	✓	✓	✓	✓	✓	✓	**8**
Implants									
Levonogestrel releasing implant: 150 mg	✓	✓	✓	✓	✗	✓	✓	✓	**7**
Etonogestrel-releasing implant:68 mg	✗	✓	✓	✓	✓	✓	✓	✓	**7**
IUDs									
Copper-containing IUD	✓	✓	✓	✓	✓	✓	✓	✓	**8**
Levonogestrel-releasing IUD	✓	✓	✓	✓	✓	✓	✓	✓	**8**
Vaginal Ring									
Progesterone-releasing vaginal ring	✗	✓	✓	✓	✓	✗	✓	✗	**5**
Emergency Contraceptives									
Ulipristal Tablet: 30 mg	✗	✓	✓	✗	✓	✓	✓	✓	**6**
Levonogestrel; 30 ug or 750 µg (pack of two); 1.5 mg	✓	✓	✓	✓	✓	✓	✓	✓	**8**
MA Medicines									
Misoprostol—mifepristone combipack	✓	✗	✓	✗	✗	✗	✗	✗	**2**
Mifepristone	✓	✗	✓	✗	✗	✗	✗	✗	**2**
Misoprostol (either 400 μg or 600 μg, PO)/200mcg/25ug^a^	✓	✓	✓	✓	✓	✓	✓	✓	**8**
**Total No. of Medicines**	**12**	**12**	**15**	**12**	**10**	**11**	**13**	**12**	

^a^Misoprostol often included in guidelines and EMLs for intrapartam care not abortion

#### Mifepristone and misoprostol

While two medical abortion medicines were listed on the current WHO EML for induced abortion (mifepristone and mifepristone-misoprostol combination regimen), misoprostol alone was listed on the WHO EML for postpartum hemorrhage (PPH). Only two countries, Afghanistan, and Lebanon, included both mifepristone-misoprostol combination regimen and mifepristone in guidelines for PPH and spontaneous abortion. Five countries (Iraq, Libya, Lebanon, Morocco, Palestine, and Pakistan) included misoprostol in guidelines for PPH and spontaneous abortion. In the updated 2023 PAC guidelines, Somalia includes Mifepristone and Misoprostol in their national guidelines. Somalia did not have PAC guidelines when the assessment was conducted.

### Inclusion of mifepristone, misoprostol, and contraceptive medicines on national EMLs

#### Contraceptives

All eight countries included ethinylestradiol-levonorgestrel (oral contraceptive) and copper-containing IUDs in both guidelines and EMLs (Table 3). In contrast to other EMR countries, Afghanistan, Iraq, Libya, and Palestine did not include all contraceptives mentioned in national family planning guidelines or their national EMLs therefore resulting in limited options for contraception. For example, in Palestine the EML included only one preventive hormonal contraception—ethinylestradiol-levonorgestrel. Although Palestine EML does not include injectables, implants and hormonal IUDs, it was noted that these medicines are available in the private sector and/or through civil and non-governmental organizations (NGOs). Levonorgestrel emergency contraceptive is mentioned in guidelines for all countries and is also listed in their national EML except for Iraq. However, it is noted in the assessment that levonorgestrel and ulipristal (emergency contraceptives) while not on the national EML in Iraq are available in the private sector.

**Table 3:** Inclusion of Essential Medicines in Essential Medicines Lists, by Country

Essential Medicines for Contraception and Medical Abortion (MA) in National Essential Medicines Lists	AFGHANISTAN	IRAQ	LEBANON	LIBYA	MOROCCO	oPt	PAKISTAN	SOMALIA	Total No. of Countries
Oral Hormonal Contraceptives									
Ethinylestradiol (30ug) + levonorgestrel (150 ug)	✓	✓	✓	✓	✓	✓	✓	✓	**8**
Ethinylestradiol (35ug) + norethisterone (1 mg)	✓	✓	✓	✓	✗	✗	✓	✓	**6**
Injectables									
Medroxyprogesterone acetate IM/SC injection	✓	✓	✓	✓	✓	✗	✓	✓	**7**
Estradiol cypionate + medroxyprogesterone acetate (5-25 mg) inj	✗	✗	✓	✗	✗	✗	✓	✓	**3**
Norethisterone enantate: 200 mg/ mL in 1- mL ampoule	✗	✗	✓	✓	✓	✗	✓	✓	**5**
Implants									
Levonogestrel releasing implant: 150 mg	✓	✗	✓	✗	✗	✗	✓	✓	**4**
Etonogestrel-releasing implant:68 mg	✗	✗	✓	✗	✓	✗	✓	✓	**4**
IUDs									
Copper-containing IUD	✓	✓	✓	✓	✓	✓	✓	✓	**8**
Levonogestrel-releasing IUD	✗	✓	✓	✗	✓	✗	✓	✓	**5**
Vaginal Ring									
Progesterone-releasing vaginal ring	✗	✗	✓	✗	✗	✗	✓	✓	**3**
Emergency Contraceptives									
Ulipristal Tablet: 30 mg	✗	✗	✓	✗	✓	✗	✗	✗	**2**
Levonogestrel; 30 ug or 750 µg (pack of two); 1.5 mg	✓	✗	✓	✓	✓	✓	✓	✓	**7**
MA Medicines									
Misoprostol—mifepristone combipack	✗	✗	✗	✗	✗	✗	✗	✗	**0**
Mifepristone	✗	✓	✗	✗	✗	✗	✗	✗	**1**
Misoprostol (either 400 μg or 600 μg, PO)/200mcg/25ug^a^	✓	✓	✓	✓	✓	✓	✓	✓	**8**
**Total No. of Medicines**	**7**	**7**	**13**	**7**	**9**	**4**	**12**	**12**	

^a^Misoprostol often included in guidelines and EMLs for intrapartam care not abortion

#### Mifepristone and misoprostol

Although two countries (Afghanistan and Lebanon) include mifepristone-misoprostol combination regimen and mifepristone in their national guidelines, none of the eight countries included them on their national EML. Mifepristone alone, however, is listed on the EML for Iraq. Iraq, however, doesn’t have a PAC or CAC guidelines. Iraq, Lebanon, Libya, Morocco, Palestine, and Pakistan, include misoprostol on EML as well as guidelines for PPH (but not for induced abortion).

### Registration of Mifepristone, Misoprostol, and contraceptive medicines in national guidelines and/or EMLs with the national regulatory authorities for marketing authorization

#### Contraceptives

As reported in Table 4, two countries didn't have an NRA that is responsible for the registration of medicines: Libya and Somalia. Libya only registers manufacturers. Most medicines on national EMLs—regardless of inclusion in guidelines—were registered for Lebanon, Morocco, Pakistan. Palestine had four drugs on national EML of which only two were registered; two additional medicines not on EML were registered as well: the emergency contraceptive ulipristal and levonorgestrel IUD. Iraq had three of the five contraceptives on the national EML registered. The most registered contraceptives were the oral contraceptive ethinylestradiol-levonorgestrel which was registered in all six countries that have a regulatory mechanism to registered medicines.

**Table 4:** Registration of Essential Medicines with National Regulatory Authority, by Country

Essential Medicines for Contraception and Medical Abortion (MA) registered with NRA	AFGHANISTAN	IRAQ	LEBANON	LIBYA	MOROCCO	oPt	PAKISTAN	SOMALIA	Total No. of Countries
Oral Hormonal Contraceptives									
Ethinylestradiol (30ug) + levonorgestrel (150 ug)	✓	✓	✓	✗	✓	✓	✓	✗	**6**
Ethinylestradiol (35ug) + norethisterone (1 mg)	✗	✗	✓	✗	✓	✗	✓	✗	**3**
Injectables									
Medroxyprogesterone acetate IM/SC injection	✓	✓	✓	✗	✓	✗	✓	✗	**5**
Estradiol cypionate + medroxyprogesterone acetate (5-25 mg) inj	✗	✗	✓	✗	✗	✗	✗	✗	**1**
Norethisterone enantate: 200 mg/ mL in 1- mL ampoule	✗	✗	✓	✗	✓	✗	✓	✗	**3**
Implants									
Levonogestrel releasing implant: 150 mg	✓	✗	✓	✗	✗	✗	✓	✗	**3**
Etonogestrel-releasing implant:68 mg	✗	✗	✓	✗	✓	✗	✓	✗	**3**
IUDs									
Copper-containing IUD	✓	✗	✗	✗	✓	✗	✓	✗	**3**
Levonogestrel-releasing IUD	✗	✓	✓	✗	✓	✓	✓	✗	**5**
Vaginal Ring									
Progesterone-releasing vaginal ring	✗	✗	✓	✗	✗	✗	✓	✗	**2**
Emergency Contraceptives									
Ulipristal Tablet: 30 mg	✗	✗	✓	✗	✓	✓	✗	✗	**3**
Levonogestrel; 30 ug or 750 µg (pack of two); 1.5 mg	✓	✗	✓	✗	✓	✓	✓	✗	**5**
MA Medicines									
Misoprostol—mifepristone combipack	✗	✗	✗	✗	✗	✗	✗	✗	**0**
Mifepristone	✗	✗	✗	✗	✗	✗	✗	✗	**0**
Misoprostol (either 400 μg or 600 μg, PO)/200mcg/25ug	✓	✓	✓	✗	✓	✓	✓	✗	**6**
**Total No. of Medicines**	**6**	**4**	**12**	**0**	**10**	**5**	**11**	**0**	

#### Mifepristone and misoprostol

No mifepristone medicine, including the combination with misoprostol, were registered in any of the eight countries. In contrast, misoprostol was registered in six countries (Afghanistan, Iraq, Lebanon, Morocco, Palestine and Pakistan).

### Procurement and forecasting of Mifepristone, Misoprostol, and contraceptive medicines in national guidelines and/or EMLs

#### Contraceptives

As reported in Table 5, despite being included in guidelines, EMLs, and/or, in some cases, registered, only Lebanon, Pakistan, and Somalia, procured and forecasted at least 10 of the 12 contraceptives. Lebanon did not procure the emergency contraceptive levonorgestrel although it was included in the country's forecasting tools. Pakistan procured levonorgestrel but not the emergency contraceptive ulipristal, which was also not included on the national EML nor registered but included in the national family planning guidelines. Iraq only procured and forecasted ethinylestradiol-levonorgestrel oral contraceptives and misoprostol; both drugs on the national EML and registered. The other contraceptives registered and included on national EML were the oral contraceptive ethinylestradiol-norethisterone, medroxyprogesterone injectable, and the levonorgestrel IUD. Almost all countries procured oral contraceptives. Five countries (Libya, Morocco, Palestine, Pakistan, and Somalia) procured the emergency contraceptive levonorgestrel, medroxyprogesterone injectable and copper IUD. Several countries, including Palestine, noted that while some medicines are not procured in the public sector, they may be procured in the private sector.

**Table 5:** Inclusion of Essential Medicines in Procurement Lists in the Last 24 Months and/or in Forecasting Tools, by Country

**Essential Medicines for Contraception and Medical Abortion (MA) in Procurement Lists and/or Forecasting Tools**	**AFGHANISTAN**^a^	**IRAQ**	**LEBANON**	**LIBYA**	**MOROCCO**	**oPt**	**PAKISTAN**	**SOMALIA**	**Total No. of Countries**
Oral Hormonal Contraceptives									
Ethinylestradiol (30ug) + levonorgestrel (150 ug)	Only Forecasting	Both	Both	Both	Both	Both	Both	Both	**7**
Ethinylestradiol (35ug) + norethisterone (1 mg)	None	None	Both	Both	None	None	Both	Both	**4**
Injectables									
Medroxyprogesterone acetate IM/SC injection	Only Forecasting	None	Both	Both	Both	Only Procurement	Both	Both	**5**
Estradiol cypionate + medroxyprogesterone acetate inj	None	None	Both	None	None	None	None	Both	**2**
Norethisterone enantate: 200 mg/ mL in 1- mL ampoule	None	None	Both	None	None	None	Both	Both	**3**
Implants									
Levonogestrel releasing implant: 150 mg	Only Forecasting	None	Both	None	None	None	Both	Both	**3**
Etonogestrel-releasing implant:68 mg	None	None	Both	None	Both	None	Both	Both	**4**
IUDs									
Copper-containing IUD	Only Forecasting	None	Both	Both	Both	Both	Both	Both	**6**
Levonogestrel-releasing IUD	None	None	Both	None	None	Only Procurement	Both	Both	**3**
Vaginal Ring									
Progesterone-releasing vaginal ring	None	None	Both	None	Only Forecasting	None	Both	None	**2**
Emergency Contraceptives									
Ulipristal Tablet: 30 mg	None	None	Both	Both	Both	Only Procurement	None	None	**3**
Levonogestrel; 30 ug or 750 µg (pack of two); 1.5 mg	Only Forecasting	None	Only Forecasting	Both	Both	Both	Both	Both	**5**
Abortion Medications									
Misoprostol—mifepristone combipack	None	None	None	None	None	None	None	None	**0**
Mifepristone	None	None	None	None	None	None	None	None	**0**
Misoprostol (either 400 μg or 600 μg, PO)/200mcg/25ug	Only Forecasting	Both	Only Forecasting	None	Both	Both	Both	Both	**5**
**Total No. of Medicines**	**0**	**2**	**11**	**6**	**7**	**4**	**11**	**11**	

^a^Afghanistan doesn't have a forecasting system

#### Mifepristone and misoprostol

No country procured mifepristone-misoprostol combination regimen. Afghanistan, Pakistan, Somalia, Morocco, Palestine, and Iraq procured misoprostol.

## Discussion

This paper describes the findings from national assessments in eight EMR countries aimed at evaluating the implementation of policy and system-level requirements necessary to ensure access to Mifepristone, Misoprostol, and contraceptives between 2020–2021.

Access to essential medicines is crucial to ensuring the right to health and achieving sustainable development. Sustainable Development Goal 3, improving human health and well-being specifically mentions “access to safe, effective, quality and affordable essential medicines and vaccines for all” (target 3.8) as an essential component of Universal Health Coverage (UHC) [21]. However, access to quality-assured and affordable medicines is challenging in many countries [22].

The WHO EMRO assessment tool to measure access to essential medicines was used to assess the situation on access to contraception and safe abortion care medicines in eight countries. The assessment tool was designed to provide information for five access measures — Guidelines, EML, Registration, Procurement, and Forecasting — across all eight EMR countries, as summarized in Table 6.

**Table 6:** Summary of inclusion of Essential Medicines, by Country

Essential Medicines for Contraception and Medical Abortion (MA)	AFGHANISTAN	IRAQ	LEBANON	LIBYA	MOROCCO	oPt	PAKISTAN	SOMALIA
**In National Family Planning and/or Abortion Care Guidelines or Portocols**								
Contraceptives	9/12	11/12	12/12	11/12	9/12	10/12	12/12	11/12
MA Medicines	3/3	1/3	3/3	1/3	1/3	1/3	1/3	1/3
**In National Essential Medicines Lists**								
Contraceptives	6/12	5/12	12/12	6/12	8/12	3/12	11/12	11/12
MA Medicines	1/3	2/3	1/3	1/3	1/3	1/3	1/3	1/3
**Registered with National Regulatory Authorities**								
Contraceptives	5/12	3/12	11/12	0/12	9/12	4/12	10/12	0/12
MA Medicines	1/3	1/3	1/3	0/3	1/3	1/3	1/3	0/3
**In Procurement Lists and/or Forecasting Tools**								
Contraceptives	0 (5^b^)/12	1/12	11 (12^b^)/12	6/12	6 (7^b^)/12	3 (6^a^)/12	10/12	10/12
MA Medicines	0 (1^b^)/3	1/3	0 (1^b^)/3	0/3	1/3	1/3	1/3	1/3

^a^Inclusion in Procurement List only

^b^Inclusion in Forcating Tools only

Increasing awareness of the availability of emergency contraceptives among women also emerged from these national assessments as an opportunity to empower women and prevent unintended pregnancy in the EMR. Emergency contraceptive levonorgestrel was procured in more countries than any other contraceptive product except for oral hormonal contraceptives. Despite this, however, many women may not be aware of this contraceptive option. According to a study in Lebanon, 75 percent of women never heard of emergency contraceptive pills [17].

### Guidelines and national essential medicine lists

The World Health Organization advocates for the use of clinical guidelines and the development and use of national essential medicine lists to promote rational use of medicines [23]. Guidelines are evidence-based recommendations intended to assist the end users to make informed decisions in a time-efficient manner “*on whether, when, and how to undertake specific actions such as clinical interventions, diagnostic tests or public health measures, with the aim of achieving the best possible individual or collective health outcomes”*[24]. The concept of essential medicine lists, including the most safe and effective medicines to meet the most important needs in a health system, was introduced by the World Health Organization in 1977 [25].

Findings from these assessments indicated that all eight countries have national family planning guidelines and most include at least one product on their national EML from each of the six contraceptive categories. However, only Lebanon, Pakistan and Somalia had all twelve contraceptives on the WHO model list of essential medicines included in their national EML. Importantly, none of these countries had developed comprehensive abortion care (CAC) guidelines and mifepristone-misoprostol combination regimen—recommended by the WHO for medical abortion—was excluded from the national EMLs and was not procured in any of these eight EMR countries.

Findings from these assessments suggest that women in EMR countries with restrictive abortion policies may encounter barriers in accessing Mifepristone and Misoprostol in the public sector and such barriers may contribute to unsafe abortions [5]. Specifically, the lack of CAC guidelines and the exclusion of mifepristone-misoprostol combination regimen from national EML hinders the availability of these medicines for women in need. While mifepristone alone was included on the EML for two countries—Iraq and Somalia—it was not registered nor procured in any country. Misoprostol is included in guidelines for the prevention of PPH in EMR countries. However, misoprostol was only procured in Afghanistan, Iraq, Palestine, Pakistan and Morocco. Therefore, even when permitted by law to preserve a woman’s life in specific countries, many women in need of abortion may not have access to Mifepristone and Misoprostol and can only rely on surgical abortion methods and procedures.

We also found that the inclusion of contraceptives in national family planning guidelines is often associated with their inclusion in national EMLs. In several countries (Iraq, Libya, and Palestine), however, nearly half of the contraceptives mentioned in their national family planning guidelines were not included on the national EML. For example, Iraq mentions emergency contraceptives—levonorgestrel and ulipristal—in family planning guidelines yet none are included in the country's EML. Therefore, efforts to expand the EML to include more contraceptive options should be considered in these countries.

### Registration

Effective and efficient regulatory systems are essential for protecting the public and enabling timely access to quality medical products [26]. Opportunities to strengthen the registration and market authorization of Mifepristone, Misoprostol, and contraceptive medicines were identified for several countries in the EMR, specifically Libya and Somalia. Access to Mifepristone, Misoprostol, and contraceptive medicines may be undermined given lack of a regulatory and registration system even when national guidelines and EMLs include these medicines. Although Somalia has developed national family planning guidelines and has an updated national EML inclusive of nearly all types of contraceptives, none of these medicines are registered. In fact, Somalia has among the lowest rates of contraceptive use globally with only 8 percent of women of reproductive age using contraceptive medicines mentioned on the EML [9] Somalia also has one of the highest rates of unintended pregnancy in the region (100 per 1,000 women and girls ages 15–49 years) of which 29 percent end in abortion [3] Therefore, ensuring the safety and effectiveness of procured contraceptives is critical in efforts to reduce unintended pregnancies in Somalia as available medicines may be substandard and falsified [27]. Efforts in regulatory system strengthening in the pharmaceutical sector are critical and needed.

Findings of these assessments provide evidence that EMR countries in the study may register Mifepristone, Misoprostol, and contraceptive medicines even if they are not included on their current national EML. Specifically, in Palestine, the emergency contraceptive ulipristal is not included on the EML yet are registered by the NRA. This suggests that the EML may need to be updated to reflect local needs and that the private sector plays an important role in the registration of contraceptives even when excluded from national EMLs. Therefore, strengthening the coordination between the public and private pharmaceutical sector may facilitate advocacy efforts to update EMLs and ensure quality and safety of medicines on the market.

### Procurement and forecasting

Alongside efforts to increase the availability of Mifepristone, Misoprostol, and contraceptive medicines registered in EMR countries, opportunities to strengthen the procurement and forecasting of these products also emerged from these national assessments. Pakistan, Somalia, and Lebanon procured the most contraceptives of all eight EMR countries. Although we found that procurement of all contraceptives and Mifepristone and Misoprostol in the public sector is lacking for all the assessed countries in the region, this does not necessarily indicate these medicines are not available or accessible in the private sector. For example, according to the assessments, Palestine and Iraq only procured oral contraceptives, injectable medroxyprogesterone acetate, ulipristal and copper IUD yet UNFPA reports indicate that both countries are more likely to use hormonal IUDs and injectable medroxyprogesterone than other EMR countries (e.g., Pakistan, Lebanon, Somalia, and Morocco) that procured them in the public sector [9].

Findings from Iraq and Palestine suggest that in some post-conflict and humanitarian settings, procurement by the private sector or by donors may be responsive to local needs and are critical to ensuring access to Mifepristone, Misoprostol, and contraceptive medicines. However, findings from Somalia and Libya suggest that in these humanitarian settings procurement from the public sector for various methods and types of contraceptives is insufficient as 81 percent of women of reproductive age have unmet need for contraception [9].

Afghanistan—which has one of the highest unintended pregnancy and maternal mortality rates globally—was the only EMR country that lacked a procurement system in the public sector from the countries assessed. Several reports indicate that women in Afghanistan may also rely on private sector and international organizations for procurement and distribution of contraceptives [14, 15]. Nearly half of women in Afghanistan have an unmet need [9] and contraceptive use is disproportionately lower than other countries in the region. Therefore, national, and international efforts to increase access to contraceptives in Afghanistan should increase the availability and distribution of these essential SRH medicines in the community, including in most in need remote areas [16].

### Strengths and limitations

This study has several strengths. First, this is the first comprehensive assessment of system-level barriers to access Mifepristone, Misoprostol, and contraceptive medicines in the EMR. Second, all eight countries utilized a standardized assessment tool which allows for cross-country comparisons. Third, the assessment incorporated both quantitative as well as qualitative approaches to data collection. For example, desktop review of policy documents was followed up with interviews with the NRA and/or MOH for each country.

Despite these strengths, there are several limitations. First, the countries’ legal and regulatory frameworks were not systematically assessed for their compatibility with the goals of universal health coverage. Second, the role of international agencies, including UNFPA, in the procurement of Mifepristone, Misoprostol, and contraceptives was not assessed. However, several countries, including Afghanistan and Somalia, mentioned UNFPA as an important partner in expanding family planning services in the country. Third, the private sector plays an important role in provision and distribution of essential medicines, including Mifepristone, Misoprostol, and contraceptive medicines, and this assessment focused on the public sector. Fourth, these assessments focused on system-level measures of access and not community-level measures of access, which includes geographic accessibility, availability, affordability, acceptability, and quality of medicines in the community. Finally, the impact of these access measures at the local level is not assessed and is critical to identifying specific barriers in accessing Mifepristone, Misoprostol, and contraceptive medicines that directly influence their use.

## Conclusion

These findings can inform efforts that aim to improve access to Mifepristone, Misoprostol, and contraceptive medicines in the EMR. Opportunities include the development of PAC and CAC guidelines, expanding national EMLs to include more options for Mifepristone, Misoprostol, and contraceptive medicines, and strengthening the registration, forecasting and procurement systems to ensure the uninterrupted availability of these essential medicines. Ministries of health may wish to consider conducting return on investments analyses to estimate lives – of mothers and newborns—and resources that would be saved with improved and effective access to contraceptive methods. Additional research and analyses are needed to identify and assess barriers to access, implementation and use before issuing new guidelines.

### Supplementary Information

Supplementary Material 1.

## Data Availability

The data used and/or analysed during the current study are available from the corresponding author on reasonable request.

## Abbreviations

CAC: Comprehensive abortion care
EML: Essential Medicines List
EMR: Eastern Mediterranean Region
NRA: National regulatory authority
PAC: Post abortion care
UNFPA: United nations Population Fund
WHO: World Health Organization
